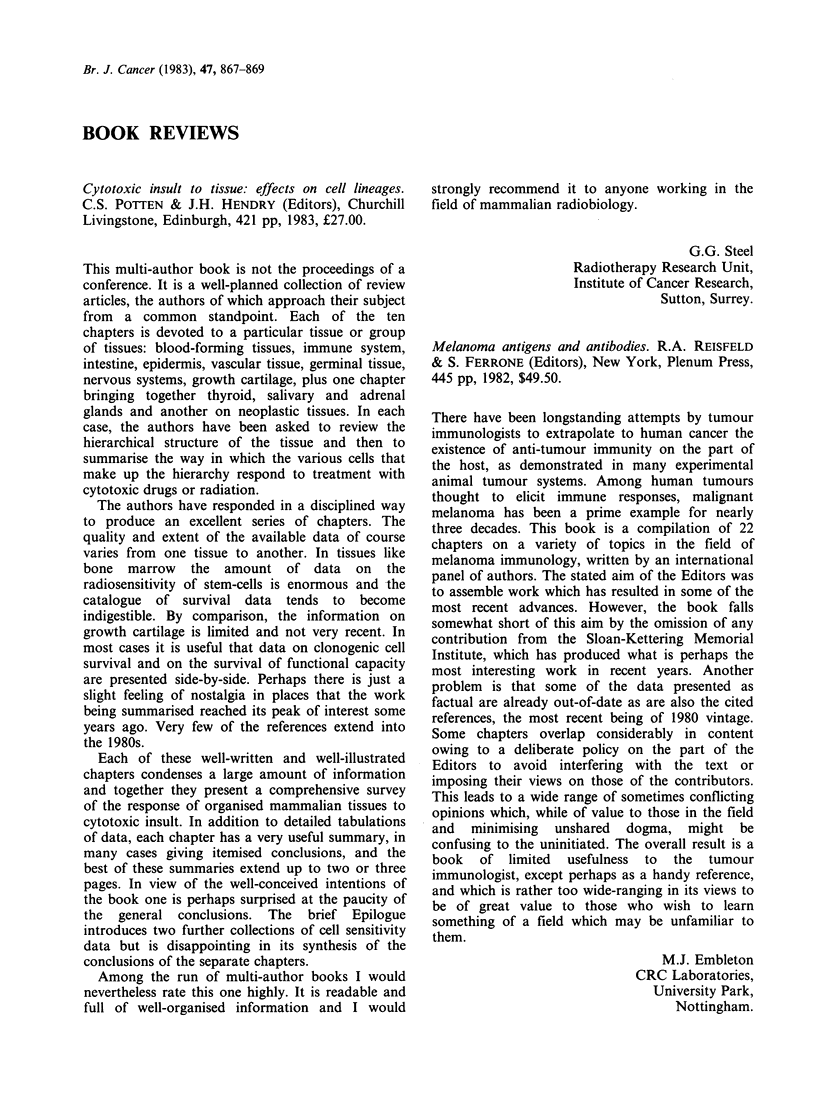# Cytotoxic insult to tissue: effects on cell lineages

**Published:** 1983-06

**Authors:** G.G. Steel


*Br. J. Cancer* (1983), **47**, 867–869

### BOOK REVIEWS

*Cytotoxic insult to tissue: effects on cell lineages.* C.S. POTTEN & J.H. HENDRY (Editors), Churchill Livingstone, Edinburgh, 421 pp, 1983, £27.00.

This multi-author book is not the proceedings of a conference. It is a well-planned collection of review articles, the authors of which approach their subject from a common standpoint. Each of the ten chapters is devoted to a particular tissue or group of tissues: blood-forming tissues, immune system, intestine, epidermis, vascular tissue, germinal tissue, nervous systems, growth cartilage, plus one chapter bringing together thyroid, salivary and adrenal glands and another on neoplastic tissues. In each case, the authors have been asked to review the hierarchical structure of the tissue and then to summarise the way in which the various cells that make up the hierarchy respond to treatment with cytotoxic drugs or radiation.

The authors have responded in a disciplined way to produce an excellent series of chapters. The quality and extent of the available data of course varies from one tissue to another. In tissues like bone marrow the amount of data on the radiosensitivity of stem-cells is enormous and the catalogue of survival data tends to become indigestible. By comparison, the information on growth cartilage is limited and not very recent. In most cases it is useful that data on clonogenic cell survival and on the survival of functional capacity are presented side-by-side. Perhaps there is just a slight feeling of nostalgia in places that the work being summarised reached its peak of interest some years ago. Very few of the references extend into the 1980s.

Each of these well-written and well-illustrated chapters condenses a large amount of information and together they present a comprehensive survey of the response of organised mammalian tissues to cytotoxic insult. In addition to detailed tabulations of data, each chapter has a very useful summary, in many cases giving itemised conclusions, and the best of these summaries extend up to two or three pages. In view of the well-conceived intentions of the book one is perhaps surprised at the paucity of the general conclusions. The brief Epilogue introduces two further collections of cell sensitivity data but is disappointing in its synthesis of the conclusions of the separate chapters.

Among the run of multi-author books I would nevertheless rate this one highly. It is readable and full of well-organised information and I would strongly recommend it to anyone working in the field of mammalian radiobiology.

G.G. Steel
Radiotherapy Research Unit,
Institute of Cancer Research,
Sutton, Surrey.